# Causal relationship between Lipdome and Chronic Obstructive Pulmonary Disease and Asthma: Mendelian randomization

**DOI:** 10.1007/s13205-024-04071-x

**Published:** 2024-09-25

**Authors:** Qiong Wu, Jingmin Fu, Cheng Zhang, Zhuolin Liu, Jianing Shi, Zhiying Feng, Kangyu Wang, Ling Li

**Affiliations:** 1 College of Humanities and Management, Hunan University of Chinese Medicine, Xueshi Road 300, Changsha, 410208 Hunan People’s Republic of China; 2 College of Traditional Chinese Medicine, Hunan University of Chinese Medicine, Xueshi Road 300, Changsha, 410208 Hunan People’s Republic of China; 3 The College of Integrated Traditional Chinese and Western Medicine, Hunan University of Chinese Medicine, Xueshi Road 300, Yuelu District, Changsha, 410208 Hunan People’s Republic of China

**Keywords:** Mendelian randomization, Lipdome, Chronic obstructive pulmonary disease, Asthma, Causal relationship

## Abstract

Genetic risk significantly influence susceptibility and heterogeneity of chronic obstructive pulmonary disease (COPD) and asthma, and increasing evidence suggests their close association with lipdome. However, their causal relationship remains unclear. In this study, we conducted a two-sample MR (Mendelian randomization) analysis using publicly available large-scale genome-wide association studies (GWAS) data to evaluate the causal impact of lipdome on COPD and asthma. The inverse variance weighted (IVW) method served as the primary analysis method, and multiple sensitivity and heterogeneity tests were performed to assess the reliability of the results. Finally, a Meta-analysis was conducted on lipdome with significant causal relationships to validate the robustness of the results. Our findings suggest that Sterol ester (27:1/18:2), Phosphatidylcholine (15:0_18:2), (16:0_18:2), (16:0_20:2), (17:0_18:2), (18:1_18:1), (18:1_18:2), (18:1_20:2), Triacylglycerol (54:3), and (56:4) levels are protective factors for COPD, while levels of Phosphatidylcholine (16:0_22:5), (18:0_20:4), and (O-16:0_20:4) are risk factors for COPD. Meta-analysis of lipids causally related to COPD also indicates significant results. Phosphatidylcholine (16:0_20:4), (16:0_22:5), and (18:0_20:4) levels are risk factors for asthma, while Phosphatidylcholine (18:1_18:2), (18:1_20:2), and Sphingomyelin (d38:1) levels are protective factors for asthma. However, the lack of statistical significance in the Meta-analysis may be due to heterogeneity in research methods and data statistics. This study indicates that 4 lipdome species have significant correlations with COPD and asthma. Phosphatidylcholine (18:1_18:2) and (18:1_20:2) are protective factors, while Phosphatidylcholine (16:0_22:5) and (18:0_20:4) are risk factors. Additionally, due to differences in molecular subtypes, phosphatidylcholine, sterol ester, and triacylglycerol exhibit differential effects on the diseases.

## Introduction

Chronic respiratory diseases (CRD) encompass diseases of the airways and other structures of the lungs, including COPD, asthma, interstitial lung disease, occupational lung diseases, and lung nodules (Gould et al. 2023). Globally, CRD accounts for a significant number of deaths, disability-adjusted life years (DALYs), incidences, and prevalence, making it a leading cause of disability and death worldwide. The healthcare costs associated with respiratory diseases continue to rise, imposing a heavy economic burden on nations and individuals (GBD Chronic Respiratory Disease Collaborators 2020; GBD 2019 Chronic Respiratory Diseases Collaborators 2023).

COPD and asthma are complex inflammatory diseases of the airways and represent the two main diseases within CRD. COPD is the leading cause of death among CRD patients, while asthma has the highest prevalence within CRD (GBD Chronic Respiratory Disease Collaborators 2020). Both asthma and COPD are heterogeneous diseases with a range of underlying mechanisms, characterized by airflow limitation (Dasgupta et al. 2023). Moreover, the pathological mechanisms of COPD and asthma are closely related to airway epithelial cell reprogramming and immune cell-mediated inflammatory responses (Miller et al. 2021; Christenson et al. 2022). However, COPD is often undiagnosed and untreated in its early stages, only receiving attention when symptoms become severe. Simultaneously, asthma has become the most common chronic disease in pediatrics, affecting adolescents and adults (Bitsko et al. 2014). The phenotypes and underlying mechanisms of COPD and asthma are not yet fully understood. It is now widely believed that asthma susceptibility has a strong genetic component (Ntontsi et al. 2021), and genetic risk plays a crucial role in the susceptibility and heterogeneity of COPD (Christenson et al. 2022).

Lipidome refers to the entire collection of chemically distinct lipid species in cells, organs, or biological systems (Kishimoto et al. 2001). Lipids are important cellular components that play critical roles in cell structure formation, cellular signal transduction, and bioenergetics (Hornburg et al. 2023). Increasing evidence suggests that dysregulation of lipid metabolism is closely associated with the pathogenesis of COPD and asthma, as it influences the occurrence and progression of these diseases (Kotlyarov and Bulgakov 2021; Loureiro et al. 2016). For example, in obese populations with high levels of triglycerides and cholesterol, poorer outcomes in COPD have been observed (Lambert et al. 2017). Cholesterol overload has been linked to COPD and airway epithelium-driven inflammation (Li et al. 2022). In asthma allergic models, sphingolipid mediators, such as sphingosine-1-phosphate and ceramide, have been shown to be important signaling molecules in airway hyperresponsiveness, mast cell activation, and inflammation (Ono et al. 2015).These different types of lipids play various roles in organisms, and among all lipid categories, mammalian cells may have thousands of individual lipid species, collectively referred to as the lipidome (Raghu 2020). Since the lipidome is composed of one or more concentric phospholipid bilayers surrounding a water core to form spherical vesicles, it has been widely used in the field of nanomedicine. Specific lipids are used as drug delivery systems, enhancing the precise targeted treatment of drugs (Cao et al. 2024). The lipidome has also been shown to be a new participant in the pathophysiology of COPD and asthma. Pulmonary surfactant is distributed on the surface of the alveolar fluid molecular layer in the lungs, with the main component being saturated phosphatidylcholine, such as phosphatidylcholine 16:0/16:0, phosphatidylcholine 16:0/14:0, and phosphatidylcholine 16:0/16:1. It can maintain the relative stability of the size of the alveolar volume (Eggers et al. 2017). Therefore, further research on the role of the lipidome in COPD and asthma may provide new insights for the comprehensive improvement of COPD and asthma management (Donnelly 2014; Ravi et al. 2021).

In the past few decades, genome-wide association studies (GWAS) have become an important research method in genetics. Mendelian randomization (MR) is a statistical method used to infer causal relationships, using summary data from GWAS to select instrumental variables (IVs) in the form of single nucleotide polymorphisms (SNPs) that meet certain conditions. These IVs are then used to explore causal relationships between exposures and outcomes (Birney 2022). Due to the random allocation of genetic variation during conception, MR reduces confounding factors and reverse causation compared to randomized controlled trials, saving a significant amount of time and effort (Davey Smith and Hemani 2014). In recent years, MR has been widely used to explore associations between various cellular molecules and a wide range of diseases. However, there is little evidence investigating the causal relationships between the lipidome and COPD or asthma. Therefore, this study aims to use large-scale GWAS data to identify potential causal relationships between the lipidome and COPD and asthma, providing new insights for the diagnosis and treatment of these conditions.

The emergence of high-sensitivity mass spectrometry has enabled people to determine an increasing number of lipid species, making lipid nomenclature both easier to manage and more descriptive (Kopczynski et al. 2020). This study provides a detailed classification of lipid species, such as phosphatidylcholine belonging to the glycerophospholipid class, and phosphatidylcholine (15:0_18:2) belonging to the molecular subtype. ‘15’ represents the number of carbon atoms, '0' indicates the number of double bonds, which are used to determine the length and saturation of fatty acyl chains (FA). (15:0_18:2) indicates that this phosphatidylcholine has two FA chains, with the second chain having 18 carbon atoms and 2 double bonds. Even within the same class of phospholipids, the structure determines the more specific functions of lipid bodies. Therefore, the results of this study further refine the analysis to the subtype level.

## Materials and methods

### Study design

In this study, we utilized significantly associated SNPs from the GWAS data of the lipidome as IVs, with COPD and asthma as outcomes. We conducted a two-sample MR analysis using summary statistics from GWAS to assess the causal relationships between the lipidome and COPD and asthma. To minimize the impact of confounding factors and avoid reverse causality, the standard MR analysis needs to meet the following three assumptions (Davies et al. 2018): (1) the IV is associated with the exposure factor; (2) the IV is independent and unrelated to any confounding factors; (3) the IV only affects the outcome through the pathway of the exposure factor. In addition, to further validate the results of the MR analysis, we performed a Mendelian randomization pleiotropy residual sum and Meta-analysis on the results with significant causal relationships.

### Data sources

The summary data for the lipidome were obtained from a recent publication on whole-genome association analysis of human plasma lipidomes. The study included 7174 individuals from the Finnish population and detected 179 lipid species belonging to 13 lipid categories using shotgun lipidomics. The four major lipid categories covered were glycerolipids, glycerophospholipids, sphingolipids, and sterols. The study conducted univariate and multivariate genome-wide analyses and identified 495 genome-wide significant loci, including 56 genetic loci (8 of which were newly identified) (Ottensmann et al. 2023).

The statistical data for COPD and asthma were retrieved from the publicly available GWAS database (https://gwas.mrcieu.ac.uk/). The COPD dataset included a total of 468,475 individuals, with 454,945 healthy controls and 13,530 COPD-positive patients. The number of SNPs in the dataset was 24,180,654. The asthma dataset included a total of 484,598 individuals, with 428,511 healthy controls and 56,087 asthma-positive patients. The number of SNPs in the dataset was 9,587,836. (COPD GWAS ID: ebi-a-GCST90018807; Asthma GWAS ID: ebi-a-GCST90038616).

### Selection of instrumental variables

To identify SNPs with significant correlations, we set the significance threshold at *P* < 1.0 × 10^–5^. However, this led to a large number of results, so we further raised the threshold. To identify strongly positive SNPs, the final threshold was set at *P* < 1.0 × 10^–8^ (Yu et al. 2023). To eliminate linkage disequilibrium, we set the clustering window at 10,000 kb and an r^2^ threshold at 0.001 to cluster SNPs and obtain independent loci. In addition, we calculated the *F*-statistic for each SNP to measure the strength of each SNP's instrumentality, with SNPs having an *F* < 10 considered weak instrumental variables (Li et al. 2023).

### MR analysis

First, we used the IVW as the primary MR method to assess the relationship between the lipidome and COPD and asthma. This method combines the Wald values for each SNP to estimate the effect, which is equivalent to weighted linear regression of the associations between instrumental variables, providing estimates unaffected by horizontal pleiotropy (Burgess et al. 2013). To further screen for significant results and account for the influence of pleiotropy, we used MR-Egger regression, weighted median, simple mode, and weighted mode as supplementary methods. The weighted median method can provide consistent estimates even with up to 50% invalid SNPs (Burgess et al. 2017), and MR-Egger can generate unbiased estimates of causal effects even when all instrumental SNPs exhibit pleiotropy (Bowden et al. 2015). Additionally, we used MR-Egger intercept to determine the presence of horizontal pleiotropy (*P* < 0.05 considered significant). Furthermore, we conducted heterogeneity analysis on the results, with *P* < 0.05 considered evidence of heterogeneity. Finally, we performed leave-one-out analysis to ensure that the MR results were not unduly influenced by individual SNPs.

### Meta-analysis of MR results

Meta-analysis is a statistical method used to compare and synthesize the results of studies on the same scientific question. By integrating all relevant studies, it provides robustness in interpreting the results. After the Meta-analysis results of MR analysis are subjected to tests for heterogeneity and multiple effects, lipid nanoparticles with more significant results are selected for Meta-analysis with relevant diseases. Cochran’s Q test is used to evaluate the heterogeneity of the results, and *P* < 0.05 indicates significant heterogeneity between the studies. In Meta-analysis, a random-effects model is chosen if there is significant heterogeneity (*P* < 0.05), otherwise (*P* > 0.05), a fixed-effects model is selected.

## Results

**Fig. 1:**
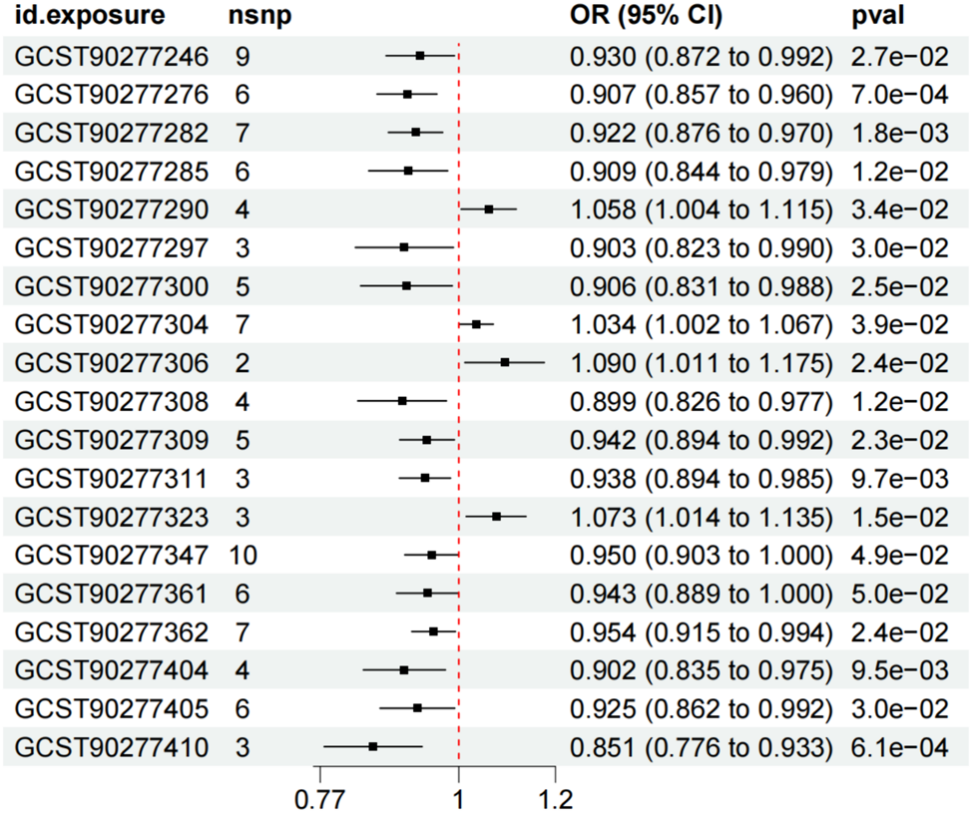
The causal relationships between 19 lipdome and COPD identified through IVW analysis, including specific exposure ID, the number of SNPs, OR value, 95% confidence interval, and p-value

**Fig. 2:**
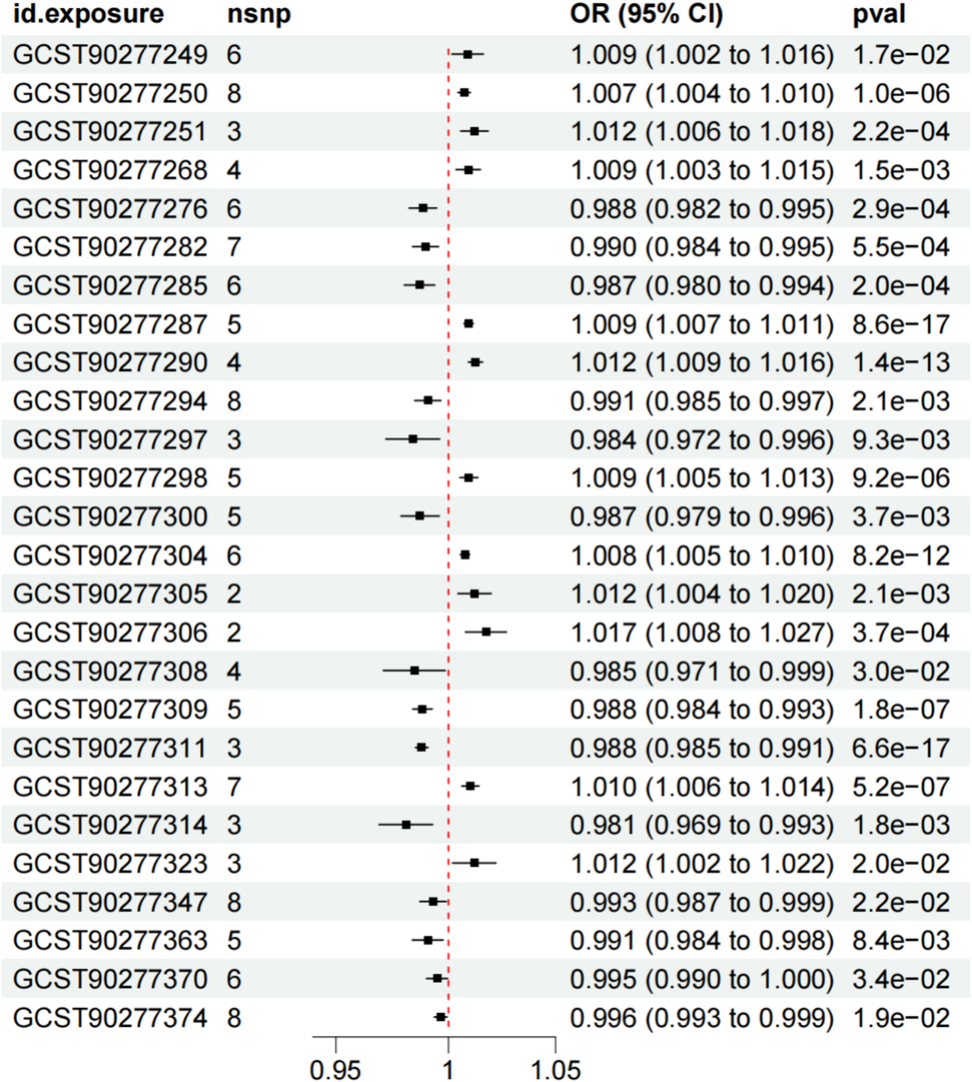
The causal relationships between 26 lipdome and asthma identified through IVW analysis, including specific exposure ID, the number of SNPs, OR value, 95% confidence interval, and p-value

Using IVW analysis as the main method, our study identified a total of 19 lipids with potential causal relationships with COPD, with four lipids associated with increased COPD risk and 15 lipids associated with decreased COPD risk (Fig. 1). In the results of asthma, a total of 26 lipids were found to have potential causal relationships, with 12 lipids associated with increased asthma risk and 14 lipids associated with decreased asthma risk (Fig. 2).

**Fig. 3:**
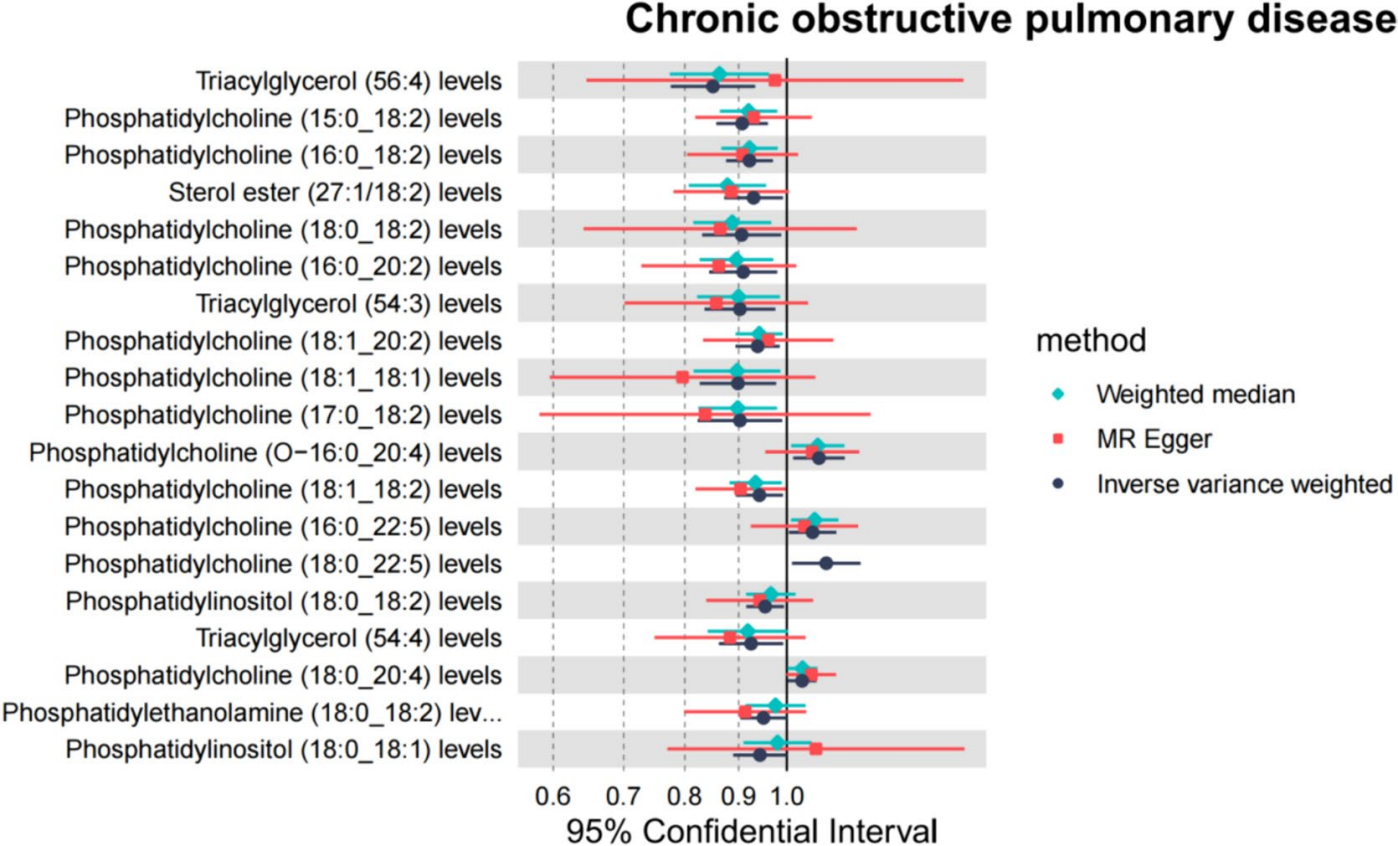
The sensitivity analysis results of lipdome and COPD include Weighted median, MR Egger and IVW

**Fig. 4:**
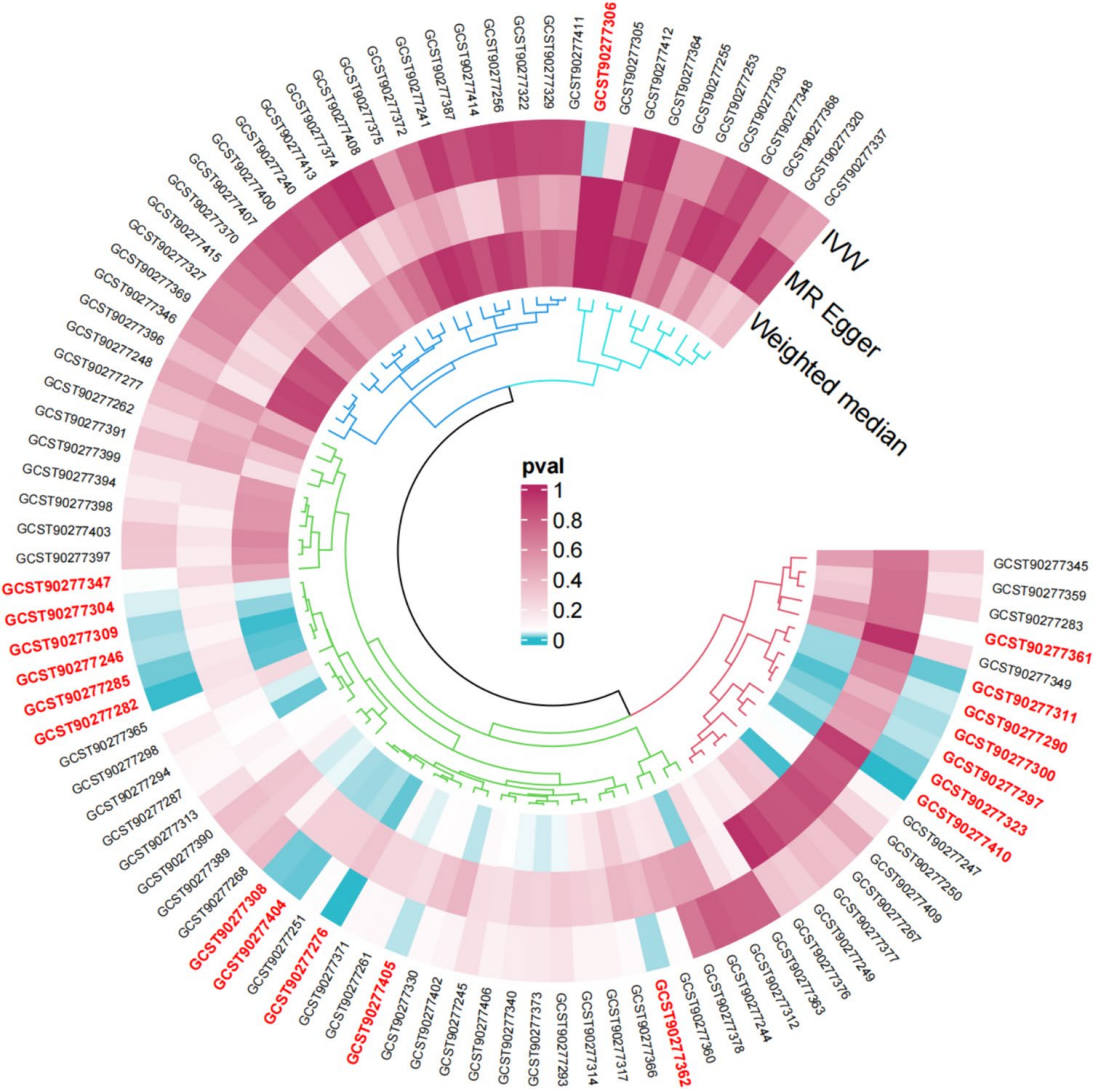
Circular plot for the sensitivity analysis of lipdome and COPD, lipdome that have significance (*Pivw* < 0.05) have been marked

**Fig. 5:**
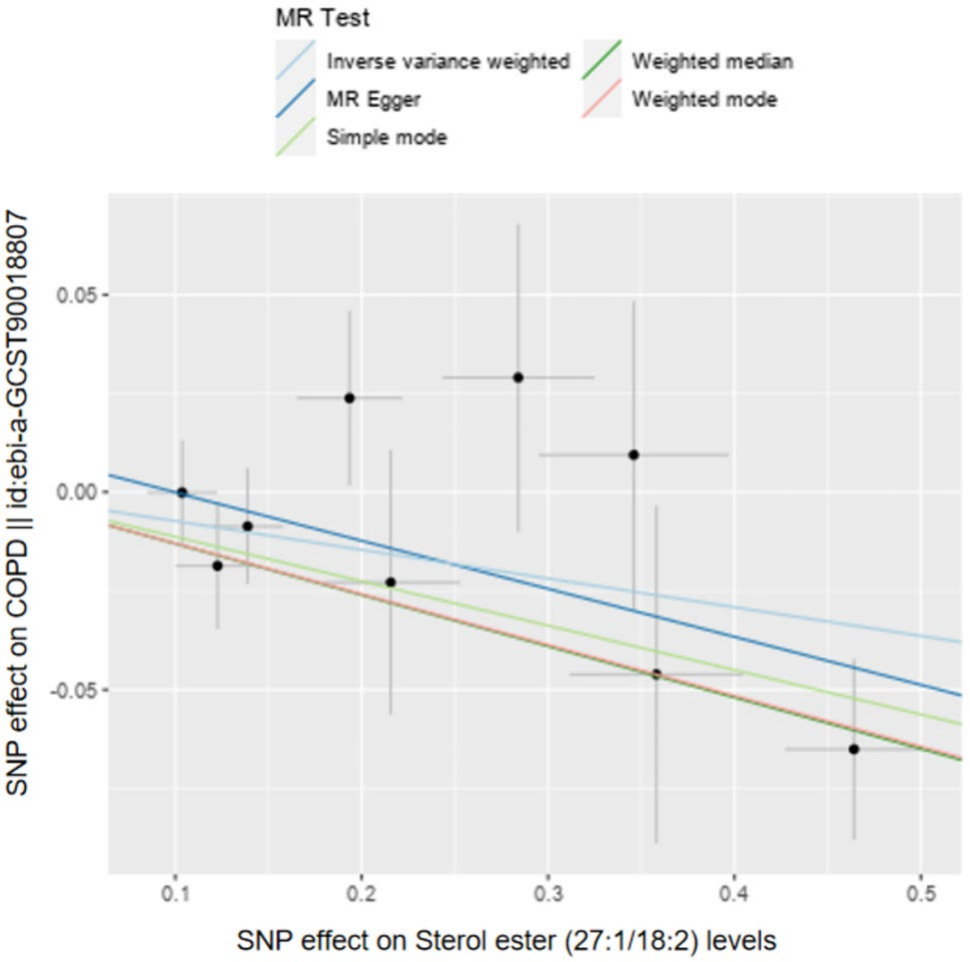
Results of the five MR analyses for Sterol ester (27:1/18:2) levels against COPD

In the sensitivity analysis of lipids and COPD (Figs. 3, 4, and Table 1), besides the IVW method, Sterol ester (27:1/18:2) levels (OR 0.930, 95% CI 0.872–0.992, *P*_ivw_ = 2.7e−02) also had a *P*-value less than 0.05 in the Weighted Median and Weighted Mode analyses, indicating a significant causal relationship with COPD and a lower risk of COPD (Fig. 5). In addition, 12 lipids showed significant causal relationships with COPD risk in both IVW and Weighted Median analyses, with *P* < 0.05, and 9 lipids were associated with a decreased risk of COPD, including: Phosphatidylcholine (15:0_18:2) levels (OR 0.907, 95% CI 0.857–0.960, *P*_ivw_ = 7.0e−04), Phosphatidylcholine (16:0_18:2) levels (OR 0.922, 95% CI 0.876–0.970, *P*_ivw_ = 1.8e−03), Phosphatidylcholine (16:0_20:2) levels (OR 0.909, 95% CI 0.844–0.979, *P*_ivw_ = 1.2e−02), Phosphatidylcholine (17:0_18:2) levels (OR 0.903, 95% CI 0.823–0.990, *P*_ivw_ = 3.0e−02), Phosphatidylcholine (18:1_18:1) levels (OR 0.899, 95% CI 0.826–0.977, *P*_ivw_ = 1.2e−02), Phosphatidylcholine (18:1_18:2) levels (OR 0.942, 95% CI 0.894–0.992, *P*_ivw_ = 2.3e−02), Phosphatidylcholine (18:1_20:2) levels (OR 0.938, 95% CI 0.894–0.985, *P*_ivw_ = 9.7e−03), Triacylglycerol (54:3) levels (OR 0.902, 95% CI 0.835–0.975, *P*_ivw_ = 9.5e−03), and Triacylglycerol (56:4) levels (OR 0.851, 95% CI 0.776–0.933, *P*_ivw_ = 6.1e−04). Three lipids were associated with increased COPD risk, including: Phosphatidylcholine (16:0_22:5) levels (OR 1.058, 95% CI 1.004–1.115, *P*_ivw_ = 3.4e−02), Phosphatidylcholine (18:0_20:4) levels (OR 1.304, 95% CI 1.002–1.067, *P*_ivw_ = 3.9e−02), and Phosphatidylcholine (O-16:0_20:4) levels (OR 1.073, 95% CI 1.014–1.135, *P*_ivw_ = 1.5e−02). The MR-Egger intercepts of all lipids were *P* > 0.05, indicating no horizontal pleiotropy. Phosphatidylcholine (18:0_22:5) levels could not be calculated for other analysis methods due to insufficient SNPs, and the other 18 lipids showed no heterogeneity. In the leave-one-out analysis, no significant abnormal SNPs were found for all lipids.

**Table 1:** Results of sensitivity analysis, heterogeneity, and horizontal pleiotropy for lipids and COPD, lipdome that have significant significance have been marked

id.exposure	GWASID	nSNP	MR Egger *P*	Weightedmedian *P*	Simple mode* P*	Weighted mode *P*	Heterogeneity tests* P*	Horizontal pleiotropy *P*
**Sterol ester (27:1/18:2)levels**	**GCST90277246**	**9**	0.101	**0.004**	0.226	0.034	0.416	0.403
**Phosphatidylcholine (15:0_18:2)levels**	**GCST90277276**	**6**	0.326	**0.013**	0.284	0.06	0.761	0.690
**Phosphatidylcholine (16:0_18:2)levels**	**GCST90277282**	**7**	0.179	**0.009**	0.264	0.056	0.700	0.798
**Phosphatidylcholine (16:0_20:2)levels**	**GCST90277285**	**6**	0.161	**0.009**	0.966	0.043	0.284	0.524
**Phosphatidylcholine (16:0_22:5)levels**	**GCST90277290**	**4**	0.586	**0.032**	0.193	0.124	0.560	0.773
**Phosphatidylcholine (17:0_18:2)levels**	**GCST90277297**	**3**	0.511	**0.010**	0.170	0.138	0.219	0.738
**Phosphatidylcholine (18:0_18:2)levels**	**GCST90277300**	**5**	0.410	**0.005**	0.432	0.056	0.190	0.766
**Phosphatidylcholine (18:0_20:4)levels**	**GCST90277304**	**7**	0.111	**0.043**	0.229	0.082	0.476	0.412
Phosphatidylcholine (18:0_22:5)levels	GCST90277306	2	0.111	/	/	/	0.723	/
**Phosphatidylcholine (18:1_18:1)levels**	**GCST90277308**	**4**	0.264	**0.024**	0.415	0.110	0.835	0.483
**Phosphatidylcholine (18:1_18:2)levels**	**GCST90277309**	**5**	0.137	**0.015**	0.287	0.075	0.788	0.405
**Phosphatidylcholine (18:1_20:2)levels**	**GCST90277311**	**3**	0.675	**0.016**	0.375	0.140	0.376	0.783
**Phosphatidylcholine (O-16:0_20:4)levels**	**GCST90277323**	**3**	0.482	**0.019**	0.304	0.167	0.896	0.793
Phosphatidylethanolamine (18:0_18:2)levels	GCST90277347	10	0.219	0.452	0.946	0.983	0.146	0.541
Phosphatidylinositol (18:0_18:1)levels	GCST90277361	6	0.721	0.061	0.688	0.723	0.462	0.494
Phosphatidylinositol (18:0_18:2)levels	GCST90277362	7	0.365	0.220	0.410	0.540	0.388	0.838
Triacylglycerol (54:3)levels	GCST90277404	4	0.271	0.024	0.568	0.112	0.512	0.640
Triacylglycerol (54:4)levels	GCST90277405	6	0.214	0.056	0.114	0.106	0.622	0.578
**Triacylglycerol (56:4)levels**	**GCST90277410**	**3**	0.922	**0.010**	0.287	0.183	0.355	0.625

**Fig. 6:**
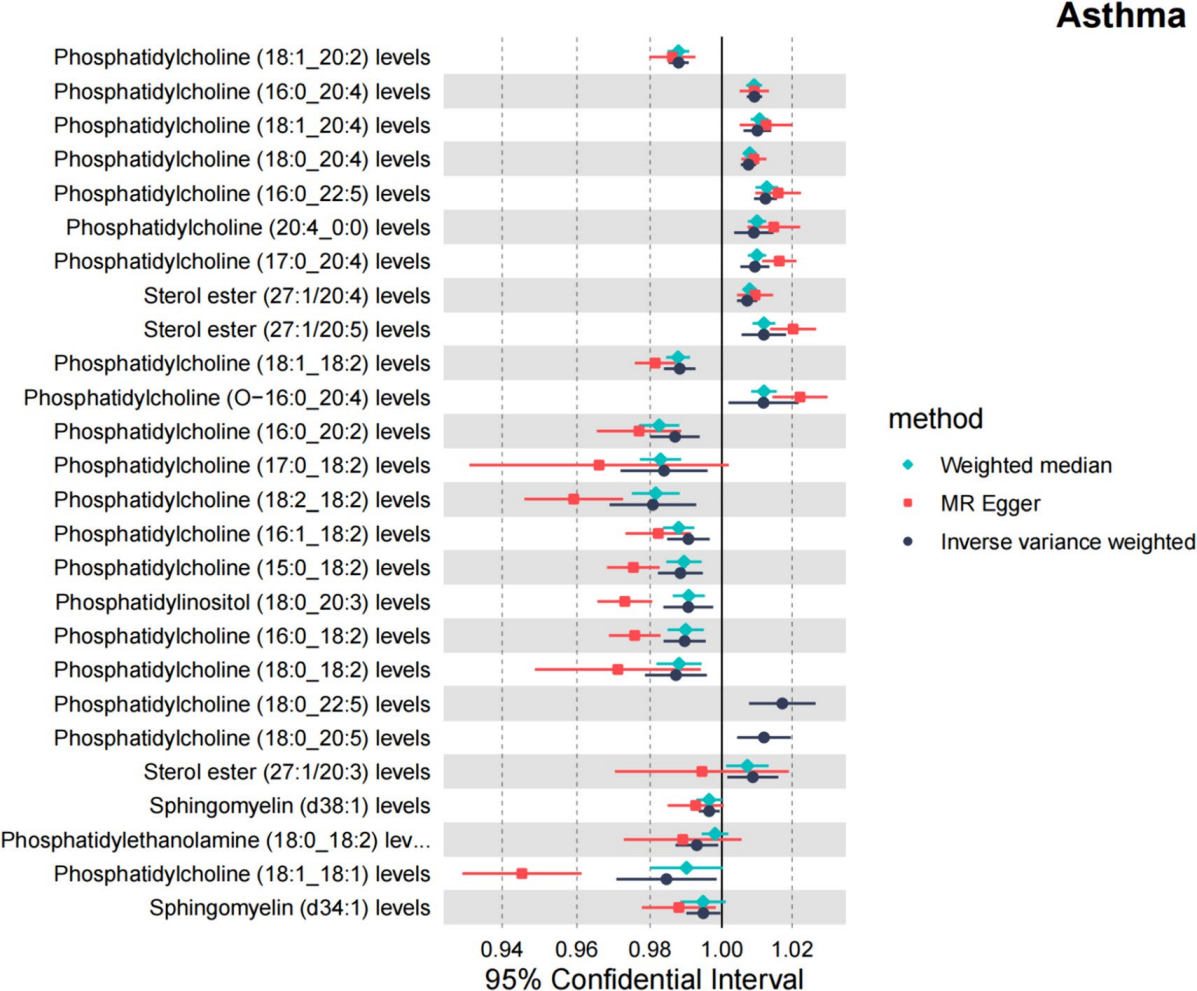
The sensitivity analysis results of lipdome and asthma include Weighted median, MR Egger and IVW

**Fig. 7:**
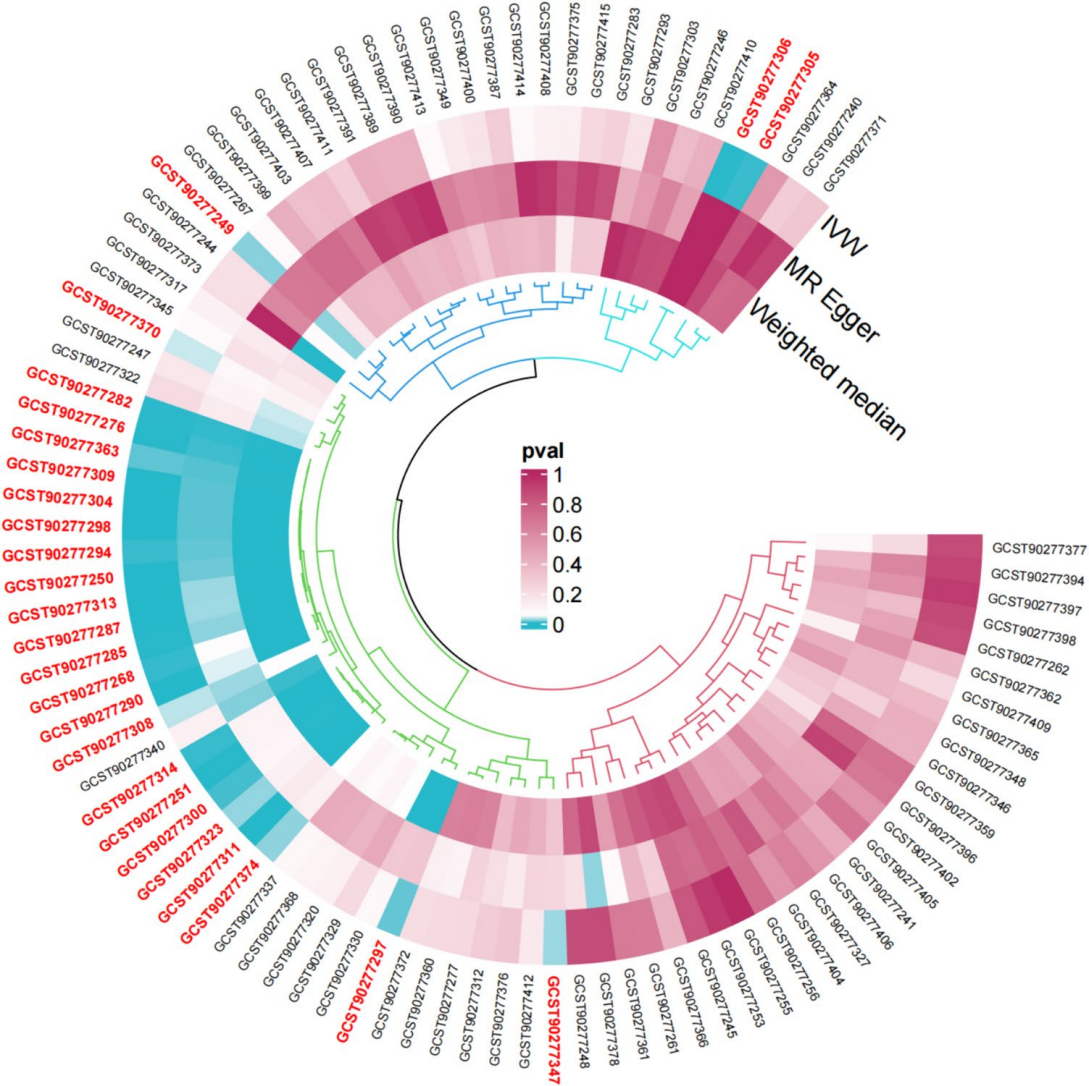
Circular plot for the sensitivity analysis of lipids and asthma, lipdome that have significance (*Pivw* < 0.05) have been marked

**Fig. 8:**
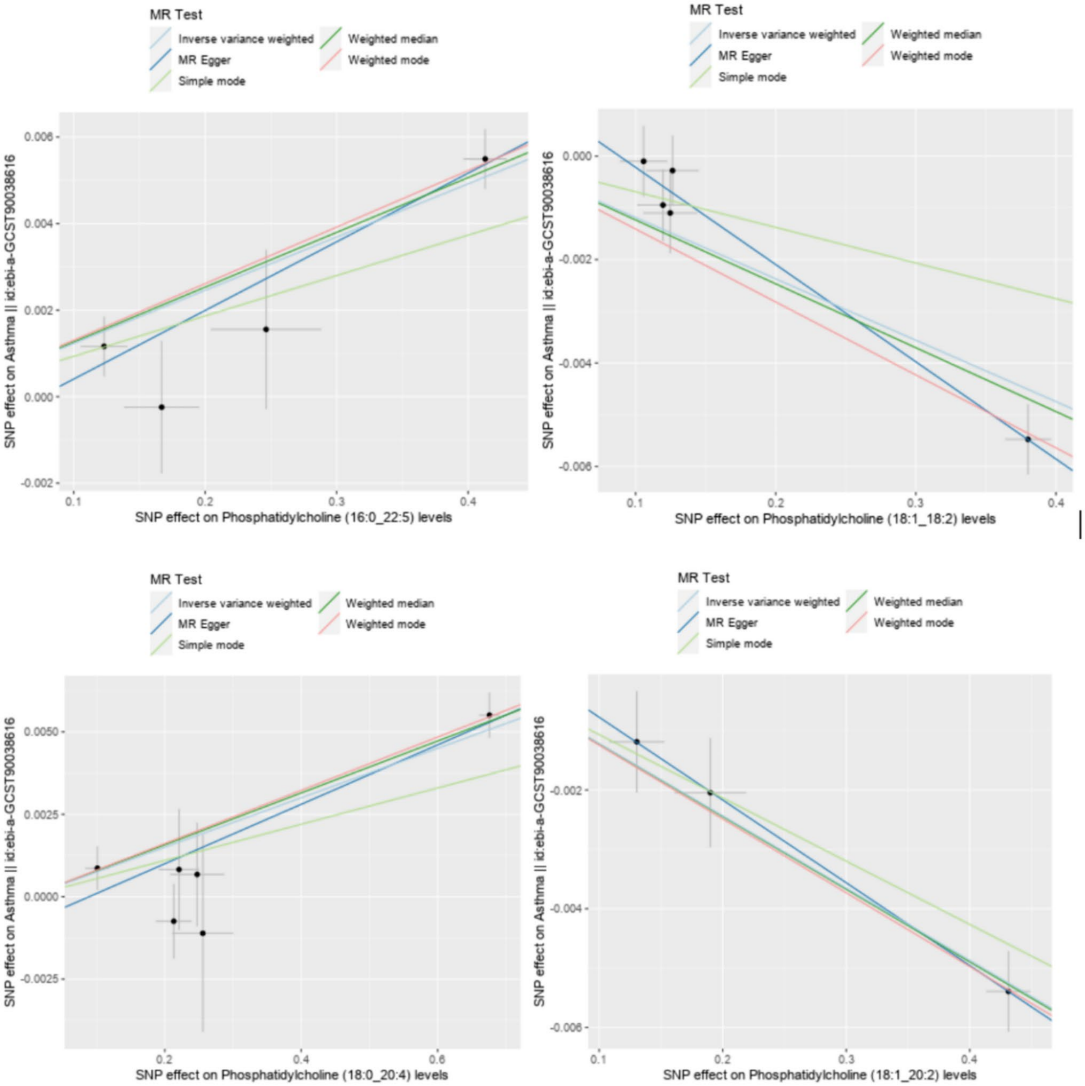
MR analysis of four lipdome with significant causal associations with asthma and COPD

**Fig. 9:**
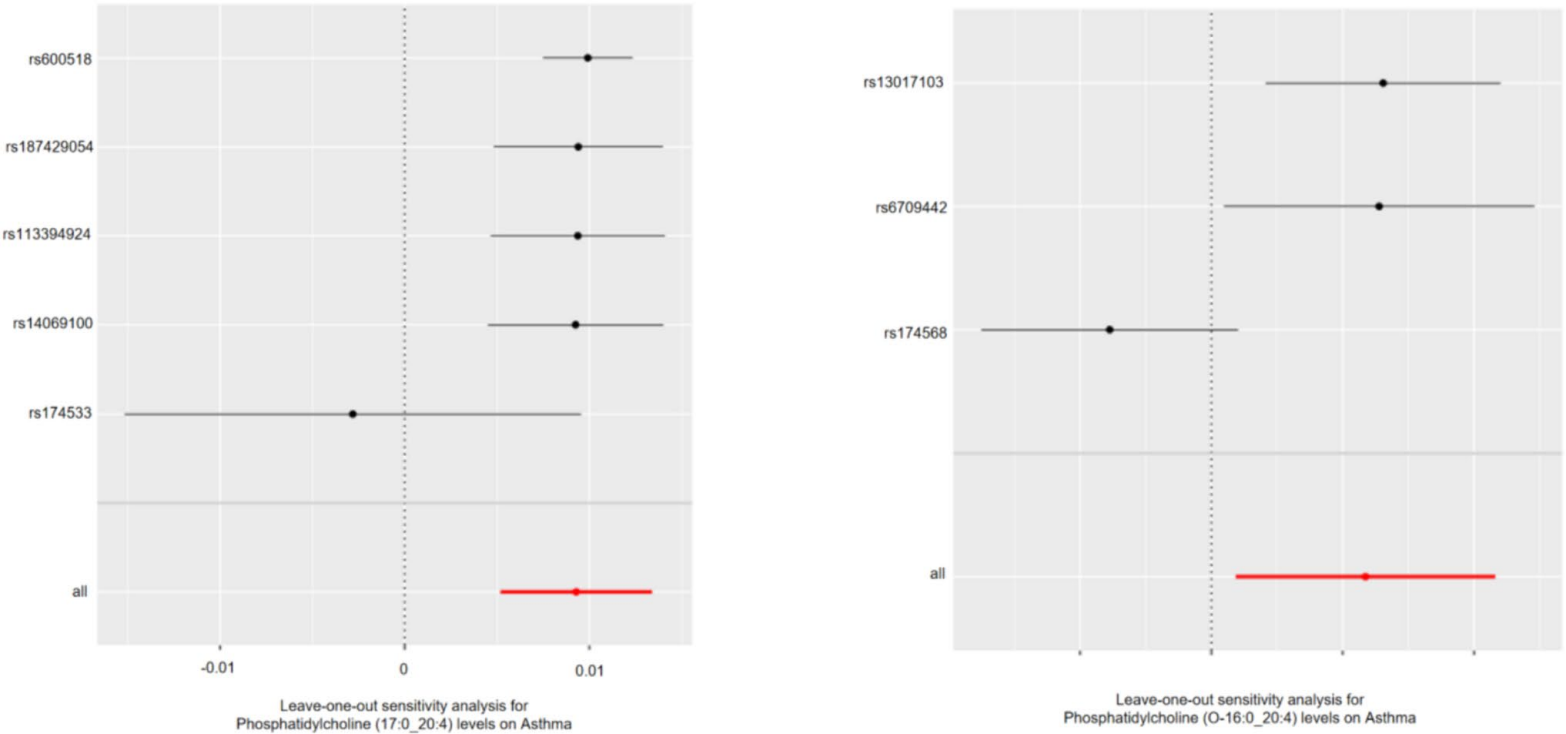
Leave-one-out analysis reveals two individual SNPs from lipids that have an impact on the outcome, SNP rs174533 having a large impact on Phosphatidylcholine (17:0_20:4) levels, and SNP rs174568 having a large impact on Phosphatidylcholine (O-16:0_20:4) levels

In the results of sensitivity analysis for lipids and asthma (Figs. 6, 7, and Table 2), besides insthe IVW method, 12 lipids showed a *P* < 0.05 in the MR Egger, weighted median, and weighted mode analyses, 9 lipids showed a *P* < 0.05 in the weighted median and weighted mode analyses, and 2 lipids showed a *P* < 0.05 in the weighted median analysis. However, among these lipids, 17 lipids had an MR-Egger intercept *P* < 0.05, indicating horizontal pleiotropy, 4 lipids showed heterogeneity, and 2 lipids did not have enough SNPs. Finally, we identified causal relationships between 6 lipids and asthma, with 4 lipids (based on 4 MR analyses with *P* < 0.05) showing a significant causal relationship with asthma (Fig. 8). These lipids include: Phosphatidylcholine (16:0_20:4) levels (OR 1.009, 95% CI 1.007–1.011, *P*_ivw_ = 8.6e−17), Phosphatidylcholine (16:0_22:5) levels (OR 1.012, 95% CI 1.009–1.016, *P*_ivw_ = 1.4e−13), and Phosphatidylcholine (18:0_20:4) levels (OR 1.008, 95% CI 1.005–1.010, *P*_ivw_ = 8.2e−12), which are associated with an increased risk of asthma, and Phosphatidylcholine (18:1_18:2) levels (OR 0.988, 95% CI 0.984–0.993, *P*_ivw_ = 1.8e−07), which is associated with a decreased risk of asthma. One lipid (based on 3 MR analyses with P < 0.05) showed a significant causal relationship with asthma and a decreased risk of asthma: Phosphatidylcholine (18:1_20:2) levels (OR 0.988, 95% CI 0.985–0.991, *P*_ivw_ = 6.6e−17). One lipid (based on 2 MR analyses with *P* < 0.05) showed a relatively significant causal relationship with asthma and a decreased risk of asthma: Sphingomyelin (d38:1) levels (OR 0.996, 95% CI 0.993–0.999, Pivw = 1.9e−02). In the leave-one-out analysis, we found that three SNPs might have a significant impact on the results, with SNP rs174581 having a large impact on Sterol ester (27:1/20:5) levels, SNP rs174533 having a large impact on Phosphatidylcholine (17:0_20:4) levels, and SNP rs174568 having a large impact on Phosphatidylcholine (O-16:0_20:4) levels (Fig. 9).

**Table 2:** Results of sensitivity analysis, heterogeneity, and horizontal pleiotropy for lipdome and asthma, lipdome that have significant significance have been marked

Id. exposure	GWAS ID	n SNP	MR Egger *P*	Weighted median *P*	Simple mode *P*	Weighted mode *P*	Heterogeneity tests* P*	horizontal pleiotropy *P*
Sterol ester (27:1/20:3) levels	GCST90277249	6	0.677	**0.017**	0.492	0.160	**0.032**	0.299
Sterol ester (27:1/20:4) levels	GCST90277250	8	**0.011**	**0.000**	0.146	**0.000**	**0.031**	0.339
Sterol ester (27:1/20:5) levels	GCST90277251	3	0.104	**0.000**	0.776	**0.019**	**0.017**	0.216
Phosphatidylcholine (20:4_0:0) levels	GCST90277268	4	0.061	**0.000**	0.050	**0.004**	**0.001**	0.215
Phosphatidylcholine (15:0_18:2) levels	GCST90277290	6	**0.003**	**0.000**	0.413	**0.001**	**0.002**	**0.018**
Phosphatidylcholine (16:0_18:2) levels	GCST90277282	7	**0.001**	**0.000**	0.393	**0.000**	**0.001**	**0.009**
Phosphatidylcholine (16:0_20:2) levels	GCST90277285	6	**0.018**	**0.000**	0.117	**0.002**	**0.005**	0.131
**Phosphatidylcholine (16:0_20:4) levels**	**GCST90277287**	**5**	**0.023**	**0.000**	0.367	**0.001**	0.400	0.976
**Phosphatidylcholine (16:0_22:5) levels**	**GCST90277290**	**4**	**0.040**	**0.000**	0.109	**0.004**	0.320	0.344
Phosphatidylcholine (16:1_18:2) levels	GCST90277294	8	**0.009**	**0.000**	0.522	**0.000**	**0.000**	0.077
Phosphatidylcholine (17:0_18:2) levels	GCST90277297	3	0.315	**0.000**	0.296	**0.019**	**0.001**	0.488
Phosphatidylcholine (17:0_20:4) levels	GCST90277298	5	**0.007**	**0.000**	0.277	**0.002**	**0.020**	0.050
Phosphatidylcholine (18:0_18:2) levels	GCST90277300	5	0.091	**0.000**	0.460	**0.003**	**0.001**	0.242
**Phosphatidylcholine (18:0_20:4) levels**	**GCST90277304**	**6**	**0.008**	**0.000**	0.236	**0.001**	0.263	0.356
Phosphatidylcholine (18:0_20:5) levels	GCST90277305	2	–	–	–	–	**0.014**	–
Phosphatidylcholine (18:0_22:5) levels	GCST90277306	2	–	–	–	–	**0.032**	–
Phosphatidylcholine (18:1_18:1) levels	GCST90277308	4	0.271	**0.046**	0.642	**0.012**	**0.000**	**0.038**
**Phosphatidylcholine (18:1_18:2) levels**	**GCST90277309**	**5**	**0.008**	**0.000**	0.297	**0.002**	0.065	0.069
**Phosphatidylcholine (18:1_20:2) levels**	**GCST90277311**	**3**	0.149	**0.000**	0.054	**0.016**	0.839	0.660
Phosphatidylcholine (18:1_20:4) levels	GCST90277313	7	**0.022**	**0.000**	0.067	**0.000**	**0.015**	0.476
Phosphatidylcholine (18:2_18:2) levels	GCST90277314	3	0.107	**0.000**	0.523	**0.021**	**0.003**	0.183
Phosphatidylcholine (O-16:0_20:4) levels	GCST90277323	3	0.113	**0.000**	0.453	**0.020**	**0.000**	0.198
Phosphatidylethanolamine (18:0_18:2) levels	GCST90277347	8	0.241	0.357	0.572	0.064	**0.000**	0.629
Phosphatidylinositol (18:0_20:3) levels	GCST90277363	5	**0.006**	**0.000**	0.460	**0.003**	**0.000**	**0.017**
Sphingomyelin (d34:1) levels	GCST90277370	6	0.085	0.097	0.091	0.891	0.466	0.217
**Sphingomyelin (d38:1) levels**	**GCST90277374**	**8**	0.117	**0.045**	0.502	0.113	0.799	0.348

**Fig. 10:**
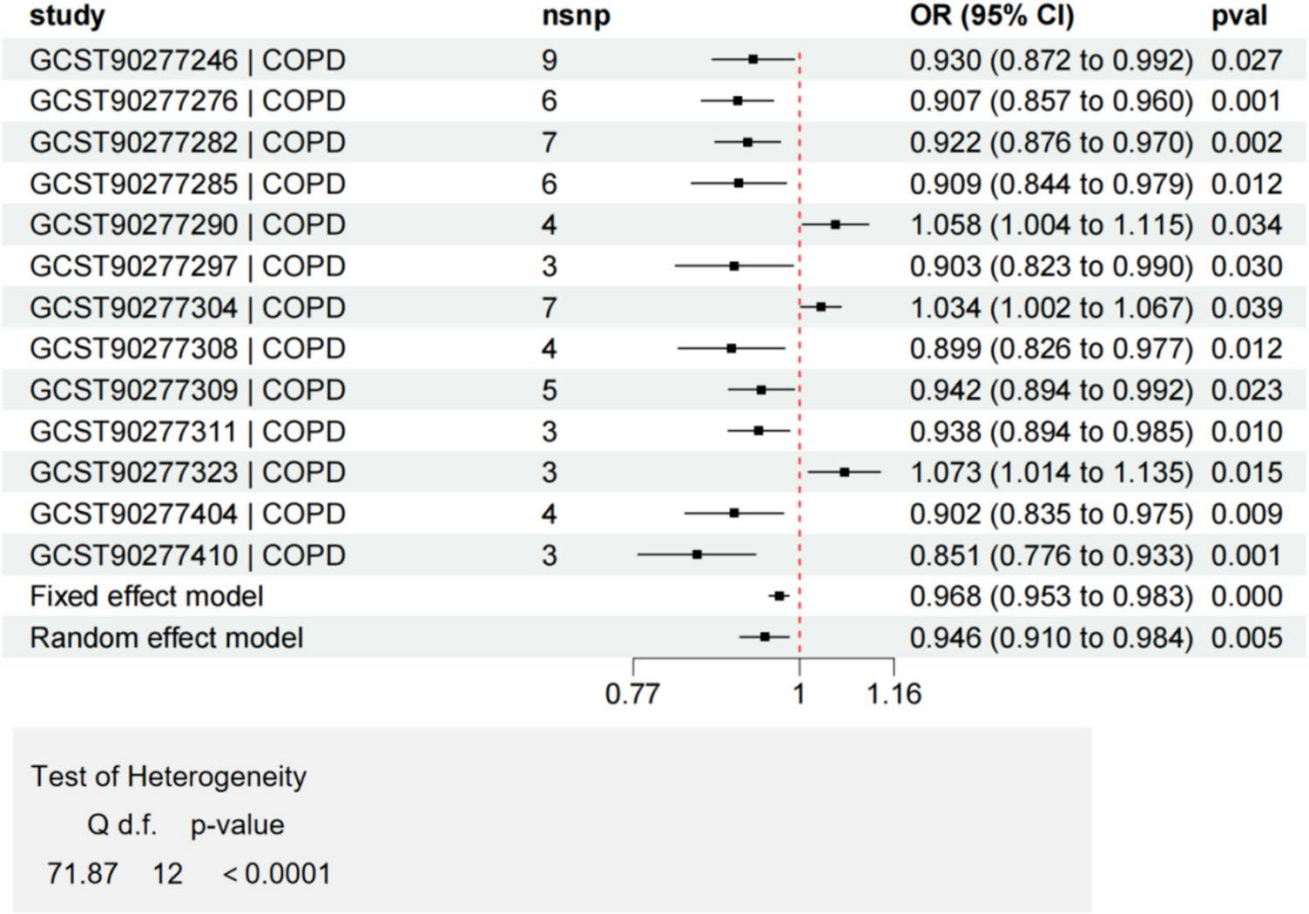
Meta analysis of lipids with significant causal associations with COPD, heterogeneity test (*P* < 0.0001)

**Fig. 11:**
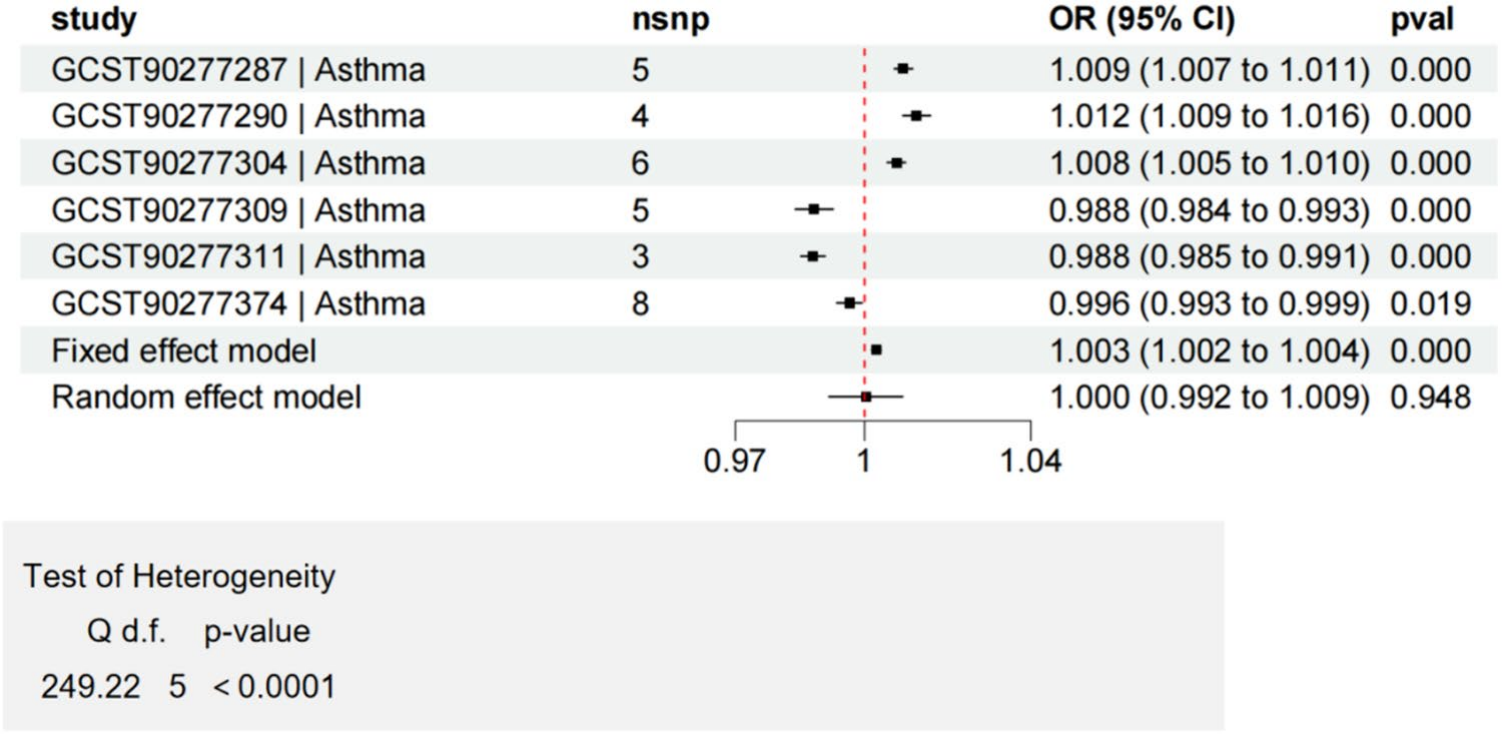
Meta analysis of lipids with significant causal associations with asthma, heterogeneity test (*P* < 0.0001)

Meta-analysis was conducted on 13 lipid components that have a causal relationship with COPD (Fig. 10). According to the results of Cochran's Q test, due to the heterogeneity (*P* < 0.0001), a random-effects model was selected, and the meta-analysis showed a significant result (*P* < 0.05), indicating a potential correlation between lipids and COPD. Similarly, a causal analysis was conducted on 6 lipid components that have a causal relationship with asthma (Fig. 11). The heterogeneity (*P* < 0.0001) also led to the selection of a random-effects model. However, the Meta-analysis with *P* = 0.948 indicated no statistical significance.

## Discussion

In this study, we conducted two-sample MR analysis using large-scale GWAS data. Our results indicate that there is a causal relationship between 13 lipid components and COPD, In this study, large-scale GWAS data were used for two-sample Mendelian randomization (MR) analysis. Our results indicate causal relationships between 13 lipid species and COPD. Sterol ester (27:1/18:2), Phosphatidylcholine (15:0_18:2), Phosphatidylcholine (16:0_18:2), Phosphatidylcholine (16:0_20:2), Phosphatidylcholine (17:0_18:2), Phosphatidylcholine (18:1_18:1), Phosphatidylcholine (18:1_18:2), Phosphatidylcholine (18:1_20:2), Triacylglycerol (54:3), and Triacylglycerol (56:4) levels are protective factors for COPD, while Phosphatidylcholine (16:0_22:5), Phosphatidylcholine (18:0_20:4), and Phosphatidylcholine (O-16:0_20:4) levels are risk factors for COPD. Meta analysis for lipid species causally associated with COPD also yielded significant results. In the MR analysis of asthma, we identified 6 lipid species that are causally related to asthma. Phosphatidylcholine (16:0_20:4), Phosphatidylcholine (16:0_22:5), and Phosphatidylcholine (18:0_20:4) levels are risk factors for asthma, while Phosphatidylcholine (18:1_18:2), Phosphatidylcholine (18:1_20:2), and Sphingomyelin (d38:1) levels are protective factors for asthma. However, the Meta analysis did not show statistical significance, which could be attributed to methodological and data heterogeneity.

Numerous previous metabolomics and lipidomics studies have indicated a correlation between lipids and chronic lung diseases. Some lipid metabolites may serve as potential biomarkers for screening, diagnosing, and treating COPD and asthma. In our study, we identified Sterol ester as the most relevant lipid component associated with COPD. As indicated in the study by Luo et al., the elevated cholesterol levels in peripheral blood are positively correlated with disease severity in patients with COPD or smokers (Luo et al. 2020). Additionally, Reed et al. found that severe COPD has been associated with increased levels of high-density lipoprotein cholesterol (Reed et al. 2011). Moreover, Sugiura et al. found that the content of cholesterol 25-hydroxycholesterol in lung tissue of COPD patients is significantly higher compared to normal individuals (Sugiura et al. 2012). Angelidis et al. discovered that cholesterol-related metabolism in lung epithelial cells and lipid fibroblasts is considered a potential marker for lung aging (Angelidis et al. 2019). However, our results indicate an inverse relationship between Sterol ester and the risk of developing COPD, which is inconsistent with the majority of previous studies, this may be related to the molecular subtype of Sterol ester. Among the lipid components associated with COPD, most of them are Phosphatidylcholine, but the differences in molecular subtypes (carbon atom number, double bond number, different positions, etc.) led to different effects of Phosphatidylcholine on COPD, Additionally, some were Triacylglycerol, indicating the need for further research to confirm the molecular subtypes of these lipid species. A study identified over 40 lipid compounds in plasma samples from COPD patients, and Phosphatidylcholine 34:3 and Triacylglycerol 52:3 were identified as potential biomarkers associated with disease severity and oxidative status in COPD (Angelidis et al. 2019). Additionally, the reduction of phospholipid and glycerol phospholipid is related to lung surfactant damage (Angelidis et al. 2019). Abnormal glycerophospholipid metabolism may be associated with the pathogenesis of non-eosinophilic subtype COPD, and the expression of lysophosphatidylcholine 18:3, lysophosphatidylethanolamine 16:1, and phosphatidylinositol 32:1 were significantly reduced in the acute exacerbation and recovery period of COPD compared to stable period (Gai et al. 2021). In a clinical study on stable chronic bronchitis in adults, inhalation of phosphatidylcholine (PC) 32:0 (the most abundant surfactant phospholipid) improved lung function in patients (Anzueto et al. 1997). A Meta-analysis showed that in the subgroup analysis of stable COPD patients who did not receive lipid-lowering therapy, their triglyceride levels were higher than those of healthy individuals (Xuan et al. 2018).

In asthma-related lipdome, the majority are Phosphatidylcholine, and there is also a type called sphingomyelin. Previous lipidomics studies have shown that the fatty acid metabolism in the bronchial epithelial cells of asthma patients is altered, leading to elevated levels of certain lipid species [phosphatidylcholine, lyso-phosphatidylcholine, and diacylglycerol phosphate], which are associated with the pathophysiology of asthma (Ravi et al. 2021). Similarly, metabolomics studies have found that elevated levels of various phosphatidylcholine and decreased levels of various lyso-phosphatidylcholine are associated with asthma (Ried et al. 2013). Furthermore, lipidomics studies have also discovered abnormal lipid metabolism in asthma patients, with significant changes in 10 lipid species in plasma, which are associated with the severity of asthma and IgE levels (Jiang et al. 2021). After inhalation of allergens, asthma patients have increased levels of oxidized phosphatidylcholine in the stimulated airways. Oxidized phosphatidylcholine may promote the pathobiology of asthma through inducing pro-inflammatory phenotypes and airway smooth muscle contraction, making it a potential new therapeutic target for oxidative stress-related pathobiology in asthma (Pascoe et al. 2021). Additionally, in asthma patients, the levels of bioactive hemolytic phosphatidylcholines 16:0 and 18:0 are significantly elevated in the lungs. The increase in hemolytic phosphatidylcholine content may be a potential key lipid mediator underlying the occurrence and progression of airway epithelial injury in asthma (Yoder et al. 2014). In a clinical study, compared to the healthy control group, asthma patients showed significantly decreased levels of sphingomyelin species (sphingomyelin 34:2, sphingomyelin 38:1, and sphingomyelin 40:1). In non-eosinophilic asthmatics, the serum levels of sphingomyelin were significantly reduced, which is consistent with our results (Guo et al. 2021). Sphingolipid metabolites are important mediators for obesity-related asthma, pediatric asthma, and exacerbation of respiratory system disease-induced airway inflammation (Guo et al. 2021). More importantly, sphingolipids have been shown to play a role in the pathogenesis of bronchopulmonary dysplasia. Interventions that interfere with sphingolipid metabolism may be a novel strategy to prevent and repair lung diseases, which could potentially reduce the incidence and mortality of severe lung diseases (Tibboel et al. 2014).

Overall, phosphatidylcholine or its subtypes show significant potential research value in COPD and asthma. More importantly, among the four lipdome identified in this study with significant implications for COPD and asthma, their impact on disease trends is consistent. Phosphatidylcholine (18:1_18:2) and Phosphatidylcholine (18:1_20:2) are protective factors, while Phosphatidylcholine (16:0_22:5) and Phosphatidylcholine (18:0_20:4) are risk factors. In recent years, the overlap in pathogenic mechanisms (such as airway inflammation), treatment methods (such as steroids), and disease gene networks between asthma and COPD suggests a high degree of similarity in the pathobiology of these two diseases (Hizawa 2024). Although phosphatidylcholine is the main component of pulmonary surfactant, research has also found significant amounts of phosphatidylglycerol [36:1], phosphatidylglycerol [34:1], and phosphatidylglycerol [34:2] in non-tumor lung tissue, all of which are components of pulmonary surfactants (Eggers et al. 2017). Therefore, the lipdome identified in this study are of great significance for the diagnosis and treatment of COPD and asthma.

This study has several strengths. The main advantage is that it is the first to use MR analysis to validate the causal relationship between lipdome and COPD and asthma, which can reduce confounding factors and reverse causality biases. Asthma is a genetically predisposed disease, and COPD has been shown to be associated with genetic risk. This study validates the relationship between lipdome and these diseases through large-scale GWAS data. MR, as an important method in genetic research, has great epidemiological significance. This study also further complements the previous lipidomics views on lung-related diseases and provides some ideas for clinical diagnosis and treatment. However, our study also has some limitations. Firstly, this study focuses on the broad scope of research on COPD and asthma, without staging COPD or classifying asthma. Secondly, all the GWAS data are derived from European populations, and whether the results are applicable to other populations needs further verification. Thirdly, in the Mendelian randomization analysis of lipdome related to asthma, the p-value we obtained did not reach statistical significance and contradicted some previous study conclusions, which may affect the accuracy of the research. Finally, although we rigorously screened the instrumental variables (significance threshold *P* < 1.0 × 10^–8^), our conclusions still need to be verified by randomized controlled experiments with high levels of evidence.

In conclusion, this study utilized publicly available large-scale GWAS data for two-sample MR analysis to assess the causal effects of lipid species on COPD and asthma. 4 lipdome species showed significant correlations with COPD and asthma. Phosphatidylcholine (18:1_18:2) and Phosphatidylcholine (18:1_20:2) are protective factors, while Phosphatidylcholine (16:0_22:5) and Phosphatidylcholine (18:0_20:4) are risk factors. Due to differences in molecular subtypes, certain phosphatidylcholine, sterol ester, and triacylglycerol also exhibit differential effects on the diseases. These complex molecular mechanisms provide further insights into the application of lipidomics in COPD and asthma.

## Data Availability

This study used publicly available GWAS data (https://gwas.mrcieu.ac.uk/), and the relevant results of this study have been included in the supplementary files.
